# COPD-OSA overlap syndrome: inflammatory and oxidative stress mechanisms, biomarkers, and clinical implications

**DOI:** 10.3389/fmed.2026.1926290

**Published:** 2026-08-18

**Authors:** Yun Xiang Cui, Qing Qing Zhang, Mao Hua

**Affiliations:** Department of Respiratory and Critical Care Medicine, Qinghai University Affiliated Hospital, Xining, China

**Keywords:** candidate biomarkers, clinical implications, COPD-OSA overlap syndrome, endothelial dysfunction, inflammation, intermittent hypoxia, oxidative stress

## Abstract

COPD-OSA overlap syndrome is associated with a greater disease burden and poorer clinical outcomes than either condition alone. COPD-related chronic airway inflammation and OSA-related intermittent hypoxia may converge to promote oxidative stress, inflammatory activation, endothelial dysfunction, and multisystem injury. This narrative mechanistic review examines these pathways using evidence from overlap syndrome, COPD, OSA, and relevant preclinical studies. It also discusses candidate biomarkers of inflammation, oxidative stress, and endothelial injury, emphasizing their exploratory status and current limitations. Established disease-directed management, phenotype-informed supportive interventions, and investigational mechanism-based strategies are reviewed. A clearer understanding of the biological and clinical heterogeneity of overlap syndrome may facilitate risk stratification, refined phenotyping, and more individualized management.

## 1 Background

Chronic obstructive pulmonary disease (COPD) and obstructive sleep apnea (OSA) are common respiratory disorders with distinct but potentially interacting pathophysiological features. COPD is characterized by persistent airflow limitation, chronic airway inflammation, cough, sputum production, and progressive dyspnea (1), whereas OSA results from recurrent upper-airway collapse during sleep, leading to intermittent hypoxia, sleep fragmentation, and sympathetic activation (2, 3). When these conditions coexist, they are referred to as COPD-OSA overlap syndrome (4). This syndrome has attracted increasing attention because it is associated with a greater clinical burden than either COPD or OSA alone (5).

Patients with overlap syndrome generally have poorer outcomes than those with either COPD or OSA alone (6, 7). Clinically, they often present with more severe nocturnal oxygen desaturation, impaired sleep quality, daytime fatigue, reduced exercise tolerance, and recurrent acute exacerbations (8). They are also at increased risk of cardiovascular and metabolic complications, including pulmonary hypertension, systemic hypertension, endothelial dysfunction, insulin resistance, and atherosclerosis (9). These observations suggest that overlap syndrome may represent a clinically important phenotype in which COPD- and OSA-related processes interact to intensify pulmonary and systemic injury.

Inflammation and oxidative stress are central mechanisms shared by COPD and OSA (10). In COPD, exposure to cigarette smoke, air pollution, biomass smoke, and recurrent airway injury activates neutrophils, macrophages, lymphocytes, and airway epithelial cells, leading to the release of inflammatory mediators such as interleukin-6 (IL-6), interleukin-8 (IL-8), tumor necrosis factor-*α* (TNF-α), and C-reactive protein (CRP) (1). Excessive reactive oxygen species production and impaired antioxidant defenses further promote epithelial injury, mucus hypersecretion, airway remodeling, and parenchymal destruction (10). In OSA, repeated cycles of hypoxia and reoxygenation activate oxidative and inflammatory signaling pathways, including nuclear factor kappa-B, while also contributing to sympathetic activation, endothelial dysfunction, metabolic dysregulation, and cardiovascular injury (2, 5). When COPD and OSA coexist, chronic airway inflammation and reduced antioxidant capacity may increase vulnerability to intermittent hypoxia-induced injury, while recurrent hypoxia–reoxygenation may further aggravate airway and systemic inflammation (3, 11).

These interacting processes have prompted interest in biomarkers that may reflect inflammatory, oxidative, and vascular injury. Candidate markers investigated in COPD, OSA, and overlap syndrome include IL-6, CRP, TNF-*α*, glutathione, superoxide dismutase, malondialdehyde, and 8-isoprostane. Their interpretation remains challenging, however, because findings vary across study populations and may be influenced by smoking, obesity, age, infection, cardiometabolic comorbidities, and medication use. In addition, many proposed molecular pathways in overlap syndrome are supported primarily by evidence from COPD or OSA alone, with limited validation in overlap-specific populations.

Recent reviews of COPD-OSA overlap syndrome have primarily addressed its prevalence, diagnosis, cardiovascular consequences, and general clinical management. However, the biological links among chronic airway inflammation, intermittent hypoxia, oxidative stress, endothelial injury, and clinical heterogeneity have not been examined within a sufficiently integrated framework. Against this background, the present narrative mechanistic review brings together overlap-specific clinical findings with relevant evidence from COPD, OSA, and preclinical research, while considering the limitations of extrapolating across these settings. By linking mechanistic pathways with candidate biomarkers, pulmonary and systemic consequences, clinical phenotypes, and therapeutic implications, this review aims to provide a more critical understanding of the biological heterogeneity of overlap syndrome and to identify priorities for future phenotyping, risk stratification, and mechanism-oriented research.

## 2 Literature identification and evidence synthesis

This narrative mechanistic review considered English-language studies published primarily between 2016 and May 2026. PubMed/MEDLINE, Web of Science, and Scopus were searched through May 2026 using terms related to COPD, OSA, overlap syndrome, intermittent hypoxia, inflammation, oxidative stress, endothelial dysfunction, biomarkers, and treatment. Reference lists of relevant original studies and recent reviews were also screened, and earlier landmark studies were included where they provided essential mechanistic or clinical context.

Overlap-specific clinical studies were prioritized. Where direct overlap-specific evidence was limited, relevant findings from COPD, OSA, and experimental studies were used to inform mechanistic interpretation. Studies were selected according to their relevance to the mechanistic, biomarker, clinical, and therapeutic scope of the review. Evidence was synthesized qualitatively, without formal meta-analysis or standardized risk-of-bias assessment, while considering study design, population characteristics, consistency of findings, potential confounding, and the directness of the evidence, including whether it was overlap-specific, indirectly derived from COPD or OSA populations, or preclinical.

## 3 Inflammatory and oxidative stress mechanisms in COPD and OSA

### 3.1 COPD: chronic airway inflammation and redox imbalance

COPD is characterized by persistent airway inflammation and progressive structural injury affecting the small airways and lung parenchyma (12). Long-term exposure to cigarette smoke and other noxious particles can disrupt epithelial integrity and activate multiple inflammatory cell populations, including neutrophils, macrophages, and lymphocytes (13). These activated cells release a broad range of mediators, such as IL-6, IL-8, and TNF-*α*, thereby sustaining a chronic inflammatory milieu (14). Persistent inflammation contributes to mucus hypersecretion, airway wall thickening, small-airway remodeling, and alveolar destruction, all of which underlie progressive airflow limitation (12).

Oxidative stress is another central feature of COPD and is closely intertwined with chronic inflammation (15). Reactive oxygen species (ROS) arise from exogenous sources, particularly cigarette smoke, as well as endogenous sources, including activated inflammatory cells, mitochondrial dysfunction, and oxidant-generating enzymes (16). Concurrently, antioxidant defense systems, including glutathione-related pathways and nuclear factor erythroid 2-related factor 2 (Nrf2)-dependent responses, may be impaired (17). The resulting redox imbalance can activate redox-sensitive pathways, including nuclear factor-κB (NF-κB), mitogen-activated protein kinase signaling, and the NLRP3 inflammasome (18). Through these processes, oxidative stress may amplify airway inflammation, accelerate tissue injury, contribute to reduced corticosteroid responsiveness, and increase susceptibility to acute exacerbations (19). These mechanisms are well described in COPD, although their relative contribution to the amplified inflammatory and oxidative burden proposed in COPD-OSA overlap syndrome requires separate evaluation.

### 3.2 OSA: intermittent hypoxia-driven oxidative and inflammatory injury

OSA is primarily caused by recurrent collapse of the upper airway during sleep (20). These repetitive obstructive events result in intermittent hypoxia-reoxygenation, sleep fragmentation, and enhanced sympathetic activation (21). Among these pathophysiological disturbances, intermittent hypoxia is considered a major driver of oxidative stress (22). Recurrent cycles of oxygen desaturation and reoxygenation increase ROS generation and produce biological effects that resemble mild, repetitive ischemia–reperfusion injury (23).

Intermittent hypoxia also activates inflammatory signaling pathways, particularly NF-κB, and promotes the release of proinflammatory mediators, including IL-6, TNF-*α*, IL-8, and CRP (21). In parallel, oxidative stress may reduce nitric oxide bioavailability and impair endothelial function (24). Together with sympathetic overactivity and sleep fragmentation, these changes contribute to systemic inflammation, vascular dysfunction, hypertension, insulin resistance, and increased cardiovascular risk in patients with OSA (25).

### 3.3 Shared and distinct pathophysiology

Although COPD and OSA arise from different initiating factors, they converge on several downstream mechanisms. Both disorders are associated with increased ROS production, activation of inflammatory signaling pathways, low-grade systemic inflammation, and endothelial dysfunction (11). These shared biological processes may help explain why both COPD and OSA are linked to cardiovascular and metabolic complications. However, the pattern of injury differs substantially between the two diseases. In COPD, inflammation and oxidative stress are typically persistent, airway-centered, and driven largely by inhaled irritants, recurrent infection, epithelial injury, and impaired tissue repair, whereas in OSA these responses are more closely related to nocturnal intermittent hypoxia, sleep fragmentation, and sympathetic activation (26). The major shared and distinct features of these mechanisms are summarized in Table 1. These shared but differently patterned processes provide the basis for the potential interactions considered in the following section.

**Table 1:** Comparative features of inflammatory and oxidative stress mechanisms in COPD and OSA and their relevance to overlap syndrome.

Feature	COPD	OSA	Relevance to overlap syndrome
Primary initiating factor	Cigarette smoke, air pollution, biomass smoke, recurrent respiratory infection	Recurrent upper-airway collapse during sleep	Chronic airway injury coexists with recurrent nocturnal intermittent hypoxia
Predominant pattern of injury	Persistent airway-centered and parenchymal inflammation	Cyclic hypoxia–reoxygenation with systemic physiological stress	Distinct injury patterns may interact synergistically
Major inflammatory cell involvement	Neutrophils, macrophages, lymphocytes, airway epithelial cells	Monocytes/macrophages, neutrophils, endothelial activation	Shared immune activation may increase the overall inflammatory burden
Principal inflammatory mediators	IL-6, IL-8, TNF-*α*, CRP	IL-6, IL-8, TNF-α, CRP	Common mediators may reflect systemic inflammation and disease activity
Main sources of ROS	Cigarette smoke, activated inflammatory cells, mitochondria, oxidant-generating enzymes	Intermittent hypoxia–reoxygenation, mitochondria, inflammatory activation	Multiple ROS-generating pathways may coexist and amplify redox injury
Antioxidant alterations	Reduced glutathione, altered SOD and catalase activity, impaired Nrf2 signaling	Evidence of impaired antioxidant defense in some studies	Reduced antioxidant reserve may increase susceptibility to oxidative damage
Key downstream consequences	Airway remodeling, emphysematous destruction, airflow limitation, exacerbations	Endothelial dysfunction, hypertension, insulin resistance, cardiovascular injury	Combined pulmonary and systemic effects may worsen clinical outcomes

Statements in the “Relevance to overlap syndrome” column represent mechanistic interpretation unless supported by direct overlap-specific clinical evidence.

## 4 Mechanistic interactions in COPD-OSA overlap syndrome

### 4.1 Potential synergistic amplification

Evidence from COPD and OSA separately supports a biologically plausible interaction between chronic airway inflammation and recurrent hypoxia-reoxygenation in COPD-OSA overlap syndrome. COPD provides a chronically inflamed and oxidant-prone background with impaired antioxidant defense (15). Persistent immune activation may further sustain this state through NF-κB-dependent cytokine signaling. When OSA is superimposed, it introduces recurrent intermittent hypoxia-reoxygenation and additional oxidative stress (27). Their coexistence may increase the overall inflammatory and oxidative burden, but most mechanistic evidence is extrapolated from COPD or OSA alone, and direct evidence of molecular synergy in overlap-specific cohorts remains limited (28). It therefore remains uncertain whether the excess clinical burden reflects true biological synergy, additive effects, or differences in disease severity and major clinical confounders.

### 4.2 Cellular and molecular mediators

#### 4.2.1 Inflammatory cell crosstalk

**Figure 1:**
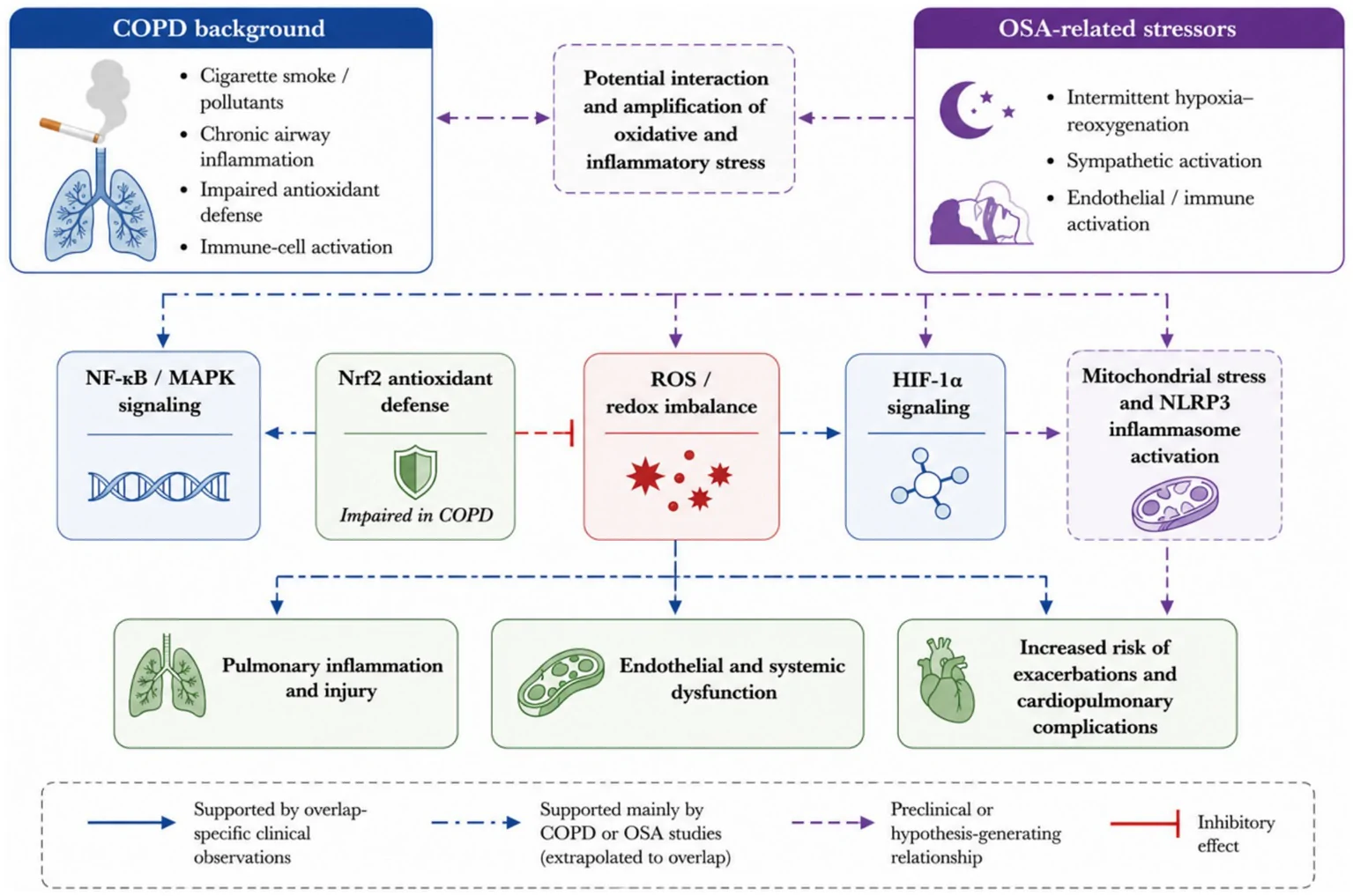
Proposed inflammatory and oxidative stress network in COPD-OSA overlap syndrome. Solid arrows denote relationships supported by overlap-specific clinical observations; dash-dot arrows indicate mechanisms supported mainly by evidence from COPD or OSA and extrapolated to overlap syndrome; dashed arrows indicate preclinical or hypothesis-generating pathways.

Multiple immune and structural cell populations implicated in COPD and OSA may collectively shape the inflammatory milieu of overlap syndrome. In COPD, neutrophils, macrophages, and airway epithelial cells sustain local inflammation through the release of cytokines, chemokines, proteases, and reactive oxygen species (16, 29). OSA-related intermittent hypoxia and sympathetic activation may additionally enhance leukocyte adhesion and endothelial activation, with reduced nitric oxide bioavailability (24, 30). However, these surrogate immune indices do not establish a specific inflammatory mechanism, and interactions among airway, circulating, and vascular compartments have not been adequately characterized in overlap-specific cohorts (Figure 1) (31).

#### 4.2.2 Redox-sensitive and hypoxia-related signaling pathways

Redox-sensitive and hypoxia-related pathways provide a plausible molecular link between COPD and OSA. Oxidative stress, cigarette smoke, infection, and intermittent hypoxia can activate NF-κB, increasing the transcription of IL-6, IL-8, TNF-*α*, and adhesion molecules (32, 33). MAPK signaling may further contribute to cytokine production, epithelial injury, and tissue remodeling (34). In parallel, impaired Nrf2-mediated antioxidant defense in COPD (29) and HIF-1α activation during OSA-related intermittent hypoxia (30) may alter the response to recurrent oxidative stress. Although these pathways are well described in the component disorders, their coordinated activation has not been demonstrated in overlap-specific populations (31).

#### 4.2.3 Mitochondrial dysfunction and inflammasome activation

Mitochondrial dysfunction and NLRP3 inflammasome activation remain largely hypothesis-generating mechanisms in COPD-OSA overlap syndrome. COPD-related cellular stress and OSA-related hypoxia–reoxygenation may increase mitochondrial reactive oxygen species production (35, 36), which may in turn promote NLRP3-dependent release of interleukin-1β and interleukin-18 (37). Direct clinical evidence remains scarce, and it is unclear whether these pathways contribute independently to disease severity or accompany a broader inflammatory and oxidative burden (38).

### 4.3 Candidate biomarker profiles

Inflammatory, oxidative, and vascular biomarkers have been explored as potential indicators of biological activity in COPD-OSA overlap syndrome, although none has been validated for routine diagnosis, risk stratification, treatment selection, or treatment monitoring (39). Candidate inflammatory markers include IL-6, CRP/hs-CRP, TNF-*α*, IL-8, and fibrinogen, which broadly reflect cytokine activation, low-grade systemic inflammation, neutrophil-related airway inflammation, and coagulation-associated inflammatory burden (40, 41). Among these, CRP/hs-CRP and fibrinogen are readily measurable in clinical practice but remain nonspecific, whereas IL-6, TNF-*α*, and IL-8 are used mainly to characterize inflammatory activity in research settings. Although higher circulating CRP or IL-6 levels have been reported in some overlap cohorts, other studies found no clear difference from COPD alone, suggesting that the observed inflammatory profile may depend partly on the comparator population and clinical characteristics of the cohort (11, 41). Interpretation is also influenced by nocturnal hypoxemia, airflow limitation, smoking, obesity, infection, cardiovascular comorbidities, medication use, and differences in the comparator populations included in individual studies (10, 39).

Markers of redox balance and vascular injury may provide complementary mechanistic information. GSH, SOD, and catalase mainly reflect antioxidant defense, whereas MDA and 8-isoprostane indicate lipid peroxidation and oxidative damage (20, 27). Endothelial and vascular markers, including ICAM-1, VCAM-1, and oxLDL, may link inflammation and oxidative stress with vascular dysfunction (42, 43). Additional exploratory markers have also been examined in overlap-specific populations. Overlap-specific comparative data are particularly limited for these markers, with much of the available evidence derived from COPD, OSA, or experimental studies. Their clinical interpretation remains limited because assay methods, sample sources, reference thresholds, and overlap-specific validation are not yet sufficiently standardized (Table 2) (27).

**Table 2:** Candidate biomarkers of inflammatory, oxidative, and endothelial injury in COPD-OSA overlap syndrome.

Biomarker	Biological domain	Mechanistic relevance	Evidence basis and current status	Major limitations/confounders
IL-6	Inflammatory cytokine	Reflects systemic cytokine activation and chronic inflammatory burden	Supported mainly by COPD and OSA studies, with limited overlap-specific data; not validated for routine clinical assessment	Obesity, infection, metabolic syndrome, cardiovascular disease
CRP/hs-CRP	Acute-phase inflammatory marker	Reflects low-grade systemic inflammation	Clinically measurable and evaluated in COPD, OSA, and some overlap cohorts, but nonspecific and unsuitable as a stand-alone overlap biomarker	Infection, obesity, vascular comorbidity, treatment status
TNF-α	Proinflammatory cytokine	Associated with immune activation, hypoxia-related inflammation, and metabolic stress	Evidence is derived primarily from COPD and OSA studies; clinical utility in overlap syndrome remains investigational	Obesity, insulin resistance, systemic inflammation
IL-8	Chemokine	Promotes neutrophil recruitment and airway inflammation	Supported mainly by COPD studies, with limited overlap-specific validation; currently used primarily for research	Smoking, infection, acute exacerbation
Fibrinogen	Inflammatory and coagulation marker	Links systemic inflammation, coagulation, and vascular risk	Clinically measurable but nonspecific; its overlap-specific prognostic value has not been established	Infection, smoking, thrombotic and vascular disease
GSH	Antioxidant marker	Reflects intracellular antioxidant defense capacity	Evidence is mainly indirect and derived from COPD, OSA, and experimental studies; currently limited to research use	Smoking, nutrition, systemic oxidative burden
SOD	Antioxidant enzyme	Limits superoxide-mediated oxidative injury	Findings from COPD, OSA, and experimental studies are variable and assay-dependent; no established clinical role in overlap syndrome	Sample source, assay variability, comorbidities, treatment
Catalase	Antioxidant enzyme	Decomposes hydrogen peroxide and contributes to antioxidant defense	Supported largely by indirect and experimental evidence; overlap-specific clinical validation is lacking	Assay variability, sample source, limited disease specificity
MDA	Lipid peroxidation marker	Indicates oxidative damage to cellular membrane lipids	Studied mainly in COPD, OSA, and experimental settings; prognostic thresholds for overlap syndrome have not been established	Smoking, diet, systemic oxidative conditions
8-Isoprostane	Oxidative stress marker	Reflects lipid peroxidation and reactive oxygen species-related injury	Remains a research marker because sampling methods and overlap-specific reference values are not standardized	Smoking, systemic oxidative injury, sample type
ICAM-1	Endothelial adhesion molecule	Reflects endothelial activation and leukocyte adhesion	Evidence comes mainly from OSA and cardiovascular studies, with limited overlap-specific data; not validated for clinical decision-making	Atherosclerosis, infection, vascular comorbidity
VCAM-1	Endothelial adhesion molecule	Reflects endothelial inflammation and vascular activation	Supported predominantly by OSA and cardiovascular evidence; its clinical relevance in overlap syndrome remains uncertain	Cardiovascular disease, metabolic disease
oxLDL	Oxidative vascular marker	Reflects lipid oxidation, endothelial injury, and atherogenic burden	Evidence is derived mainly from OSA and cardiometabolic studies; not validated as an overlap-specific prognostic marker	Dyslipidemia, metabolic syndrome, atherosclerosis

CRP, C-reactive protein; hs-CRP, high-sensitivity C-reactive protein; GSH, glutathione; SOD, superoxide dismutase; MDA, malondialdehyde; ICAM-1, intercellular adhesion molecule-1; VCAM-1, vascular cell adhesion molecule-1; oxLDL, oxidized low-density lipoprotein.

No biomarker has yet been validated for routine overlap-specific diagnosis, risk stratification, treatment selection, or treatment monitoring.

At present, no reproducible biomarker profile has been shown to distinguish overlap syndrome reliably from its component disorders or to predict prognosis (11, 41). Biomarker interpretation should therefore be integrated with clinical and physiological variables, including nocturnal oxygen burden, apnea–hypopnea index, pulmonary function, exacerbation history, body mass index, smoking status, and cardiovascular comorbidities (39). Future studies should examine whether multimarker profiles combined with physiological and clinical phenotyping improve the prediction of exacerbations, cardiovascular events, treatment response, and mortality. Until such approaches are prospectively validated, these biomarkers are better suited to biological characterization and hypothesis generation than to routine clinical decision-making (44, 45).

## 5 Pulmonary dysfunction and systemic consequences

### 5.1 Pulmonary consequences

#### 5.1.1 Airway, parenchymal, and gas-exchange impairment

In COPD-OSA overlap syndrome, pulmonary impairment is determined largely by the underlying COPD, including persistent airflow limitation, small-airway dysfunction, parenchymal destruction, and impaired gas exchange (46, 47). Coexisting OSA adds recurrent upper-airway obstruction during sleep and may further reduce ventilatory reserve, particularly in patients with advanced airflow limitation or baseline hypoxemia (48). Pulmonary dysfunction can be characterized by FEV1, the FEV1/FVC ratio, MMEF, and resting or nocturnal oxygenation. However, direct evidence that coexisting OSA causes additional structural lung injury beyond that attributable to COPD remains limited (49).

#### 5.1.2 Nocturnal hypoxemia and exacerbation vulnerability

More severe and prolonged nocturnal hypoxemia is one of the most clinically relevant features of COPD-OSA overlap syndrome. In patients with COPD, baseline ventilation–perfusion mismatch and limited ventilatory reserve may already compromise oxygenation during sleep. Superimposed OSA introduces recurrent upper-airway collapse, thereby increasing the depth and frequency of nocturnal desaturation (50, 51). This greater hypoxic burden may contribute to morning symptoms, reduced exercise tolerance, and diminished physiological reserve (52, 53). Observational studies also suggest greater vulnerability to acute exacerbations and hospitalization, although the extent to which this risk is independently attributable to OSA remains uncertain because COPD severity, obesity, cardiometabolic disease, and treatment adherence may also influence outcomes (54).

### 5.2 Systemic consequences

#### 5.2.1 Cardiovascular and pulmonary vascular injury

Patients with overlap syndrome may have a greater cardiovascular and pulmonary vascular burden, including systemic hypertension, pulmonary hypertension, arrhythmia, ischemic heart disease, and heart failure (3, 46, 54). Nocturnal hypoxemia, sympathetic activation, and endothelial dysfunction are plausible contributors, but their relative importance and the independent effect of coexisting OSA remain uncertain. Pulmonary hypertension may further reduce right ventricular function and overall cardiopulmonary reserve.

#### 5.2.2 Metabolic and functional consequences

**Figure 2:**
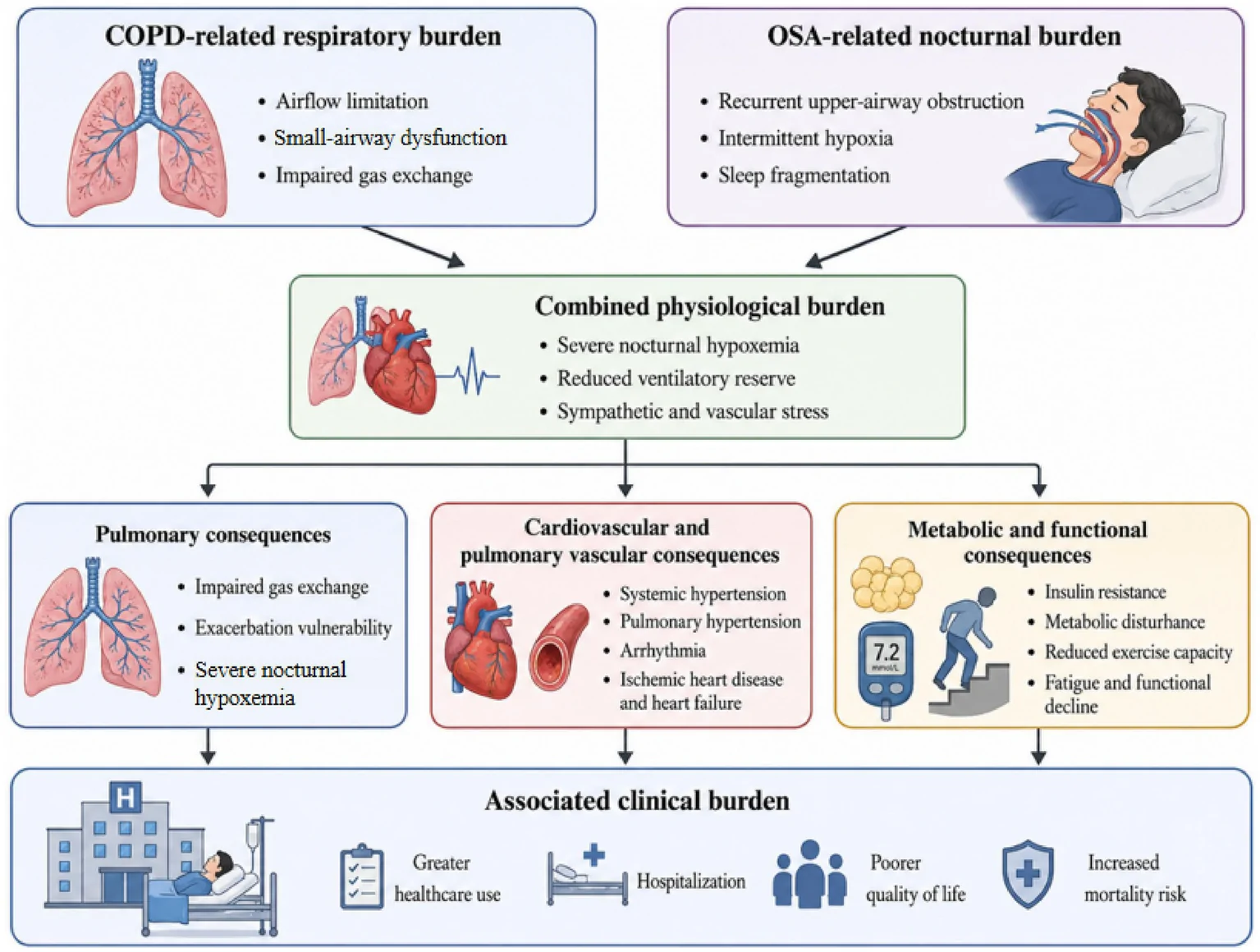
Pulmonary and systemic consequences associated with COPD-OSA overlap syndrome. The figure summarizes the principal clinical manifestations associated with combined airflow limitation, nocturnal hypoxemia, and reduced cardiopulmonary reserve, including impaired gas exchange, exacerbation vulnerability, pulmonary vascular disease, cardiovascular complications, metabolic disturbance, and functional decline. Arrows indicate conceptual associations rather than established causal pathways.

Metabolic and functional impairment represents another important dimension of the clinical burden in overlap syndrome. OSA may contribute through insulin resistance, altered glucose metabolism, and adipose tissue inflammation, whereas COPD may contribute through physical inactivity, skeletal muscle dysfunction, and, in some patients, corticosteroid exposure (52). Obesity and nocturnal hypoxemia may compound these risks, although the extent of any overlap-specific effect remains uncertain (35). Functional decline is likely multifactorial, reflecting pulmonary limitation, impaired gas exchange, cardiovascular impairment, muscle deconditioning, sleep fragmentation, and daytime somnolence (55). These clinical domains are summarized in Figure 2.

### 5.3 Clinical implications

The coexistence of COPD and OSA may increase symptom and healthcare burden, particularly when severe airflow limitation is accompanied by marked nocturnal oxygen desaturation. However, dyspnea, fatigue, poor sleep quality, daytime somnolence, and exercise intolerance are nonspecific and may also reflect obesity, cardiovascular disease, chronic hypercapnia, or medication effects.

Assessment should therefore extend beyond spirometry and the apnea-hypopnea index to include nocturnal oxygen burden, ventilatory status, exacerbation history, positive airway pressure adherence, cardiovascular and metabolic comorbidities, exercise capacity, and health-related quality of life. Reported associations with hospitalization and mortality vary across studies and should be interpreted in light of diagnostic criteria, disease severity, cohort characteristics, and treatment exposure. Representative assessment domains are summarized in Table 3.

**Table 3:** Major clinical manifestations and assessment domains in COPD-OSA overlap syndrome.

Domain	Major manifestations	Representative indices/outcomes
Pulmonary function	Airflow limitation, small-airway dysfunction, parenchymal injury, impaired gas exchange	FEV1, FEV1/FVC, MMEF, and resting oxygenation
Sleep-related respiratory burden	Severe nocturnal hypoxemia, reduced ventilatory reserve, possible nocturnal hypoventilation	Overnight oximetry, oxygen desaturation burden, sleep-related oxygen indices
Acute clinical deterioration	COPD exacerbations, emergency visits, hospitalization	Exacerbation frequency, emergency attendance, hospital admission
Cardiovascular and pulmonary vascular consequences	Systemic hypertension, pulmonary hypertension, arrhythmia, ischemic heart disease, heart failure	Blood pressure, echocardiographic findings, cardiovascular events
Metabolic and functional consequences	Insulin resistance, metabolic disturbance, reduced exercise capacity, functional decline	BMI, glucose-related indices, exercise tests, activity level
Patient-centered and prognostic outcomes	Greater symptom burden, poorer quality of life, hospitalization, mortality	Symptom scores, quality-of-life measures, healthcare use, survival

## 6 Therapeutic implications: established management and investigational mechanism-based strategies

The interplay among chronic airway inflammation, oxidative stress, and intermittent hypoxia in COPD-OSA overlap syndrome suggests that management should address both lower-airway disease and sleep-disordered breathing. Established COPD- and OSA-directed therapies should remain the foundation of care, whereas lifestyle measures and mechanism-based adjuncts should be interpreted according to their level of evidence. In particular, antioxidant, anti-inflammatory, and pathway-targeted strategies remain investigational rather than established treatments.

### 6.1 Established disease-directed management

#### 6.1.1 COPD-directed treatment

Standard COPD therapies remain essential in overlap syndrome. Long-acting bronchodilators improve airflow limitation, reduce hyperinflation, and relieve dyspnea, thereby improving ventilatory mechanics and functional capacity (55). In selected patients, especially those with frequent exacerbations or eosinophilic features, inhaled corticosteroid-containing regimens may reduce exacerbation risk and inflammatory burden (56). Their use should nevertheless follow established COPD indications rather than the presumed inflammatory burden of overlap syndrome itself. Smoking cessation is also important because it removes a major source of oxidant exposure and chronic inflammatory stimulation (50). Pulmonary rehabilitation may further improve exercise tolerance, muscle function, and health status (57). These interventions target the lower-airway component of overlap syndrome, but they do not directly address OSA-related intermittent hypoxia (53).

#### 6.1.2 OSA-directed treatment

OSA-directed therapy is equally important because recurrent upper-airway obstruction and intermittent hypoxia may amplify oxidative stress and vascular injury in overlap syndrome (48). Continuous positive airway pressure (CPAP) remains the principal therapy for most patients with OSA and can reduce nocturnal obstruction, intermittent hypoxia, and sleep fragmentation. Through these effects, CPAP may lessen sympathetic activation, endothelial dysfunction, and systemic inflammatory or oxidative stress responses (50). However, evidence for corresponding changes in circulating inflammatory and oxidative stress markers remains inconsistent, and overlap-specific data are limited. Most biomarker studies have been conducted in OSA populations, with variable responses reported after CPAP. These variations may reflect differences in treatment adherence, obesity, baseline disease severity, biomarker selection, and duration of follow-up. Changes in biomarker levels should therefore not be used as a routine measure of treatment response in overlap syndrome. In selected patients, bilevel positive airway pressure or other noninvasive ventilatory support may be considered, particularly when nocturnal hypoventilation, chronic hypercapnia, or ventilatory insufficiency is present (53). Clinical assessment should remain focused on adherence, nocturnal oxygenation, sleep-related symptoms, daytime function, and ventilatory status.

#### 6.1.3 Importance of combined management

**Figure 3:**
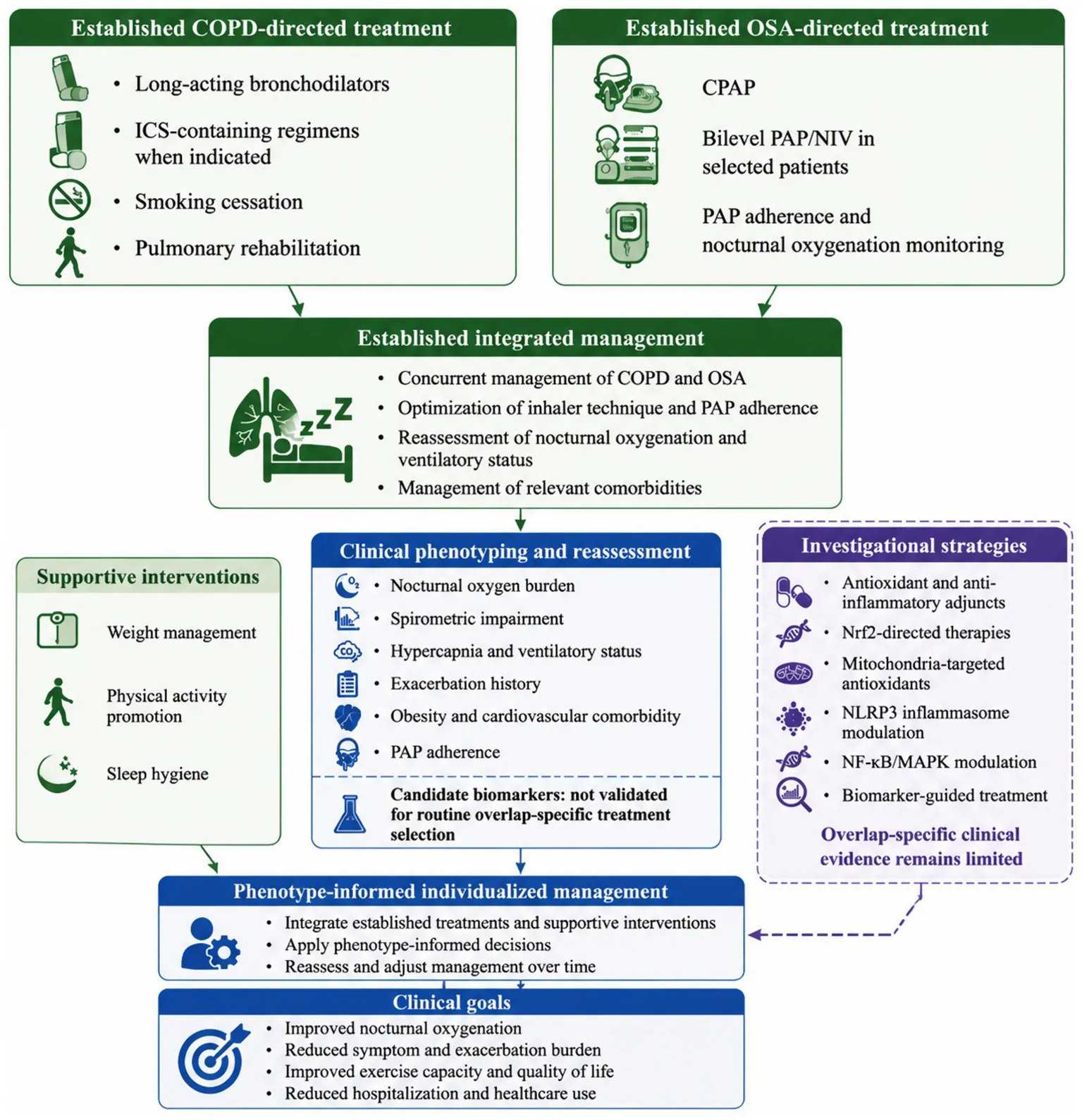
Evidence-positioned management framework for COPD-OSA overlap syndrome. Established COPD- and OSA-directed therapies form the basis of integrated management, supported by lifestyle interventions, clinical phenotyping, and repeated assessment. Pathway-targeted and biomarker-guided strategies remain investigational because overlap-specific clinical evidence is limited. Solid arrows represent current management pathways, whereas dashed arrows indicate approaches requiring further clinical validation.

Because COPD and OSA contribute distinct but interacting pathophysiological drivers, treating only one component may be insufficient. COPD-directed therapy can improve airflow limitation and baseline respiratory vulnerability, but it does not eliminate OSA-related intermittent hypoxia (39). Conversely, positive airway pressure improves sleep-disordered breathing but does not directly reverse chronic airway remodeling, mucus hypersecretion, or smoking-related lung injury (48). Therefore, integrated COPD- and OSA-directed management forms the basis of treatment for overlap syndrome. By addressing lower-airway disease and sleep-disordered breathing concurrently, this approach provides more comprehensive management than treatment directed at either condition alone (Figure 3) (5). Its clinical effects are likely to vary according to COPD phenotype, OSA severity, nocturnal hypoxemic burden, obesity, hypercapnia, cardiovascular comorbidity, and adherence to positive airway pressure, all of which should be considered during treatment selection and follow-up.

### 6.2 Supportive and phenotype-relevant adjunctive strategies

#### 6.2.1 Lifestyle and non-pharmacological interventions

Lifestyle and non-pharmacological measures may complement standard COPD- and OSA-directed therapy, particularly in selected phenotypes. Weight management may be beneficial in patients with obesity, as excess adiposity can worsen upper-airway collapsibility, systemic inflammation, insulin resistance, and cardiometabolic risk (55, 58). Exercise training may improve physical conditioning, skeletal muscle function, and exercise tolerance, while reducing inactivity-related functional decline (59). Sleep hygiene and optimization of sleep-related behaviors may also support treatment adherence and symptom control. Although these interventions are not specific to overlap syndrome, they may modify important contributors such as ventilatory load, metabolic dysfunction, systemic inflammation, and oxidative stress (60).

#### 6.2.2 Potential antioxidant and anti-inflammatory adjuncts

Antioxidant and anti-inflammatory approaches have attracted interest because oxidative stress and persistent inflammatory activation are common to both COPD and OSA. Conventional antioxidant strategies have been studied mainly in either condition, but their effects on oxidative biomarkers and clinical outcomes have been inconsistent, with little evidence available in overlap syndrome. It therefore remains unclear whether reducing systemic oxidative stress improves nocturnal oxygenation, exacerbation risk, cardiovascular outcomes, or functional status. Anti-inflammatory treatment raises similar concerns. Inhaled corticosteroids and other established COPD therapies should continue to be prescribed according to conventional COPD indications rather than as nonspecific treatment for systemic inflammation. Although more selective modulation of inflammatory pathways may be biologically plausible, broad suppression of inflammation may increase susceptibility to respiratory infection, and its benefit–risk balance has not been established in overlap-specific studies (61–63).

### 6.3 Investigational pathway-targeted and precision-oriented directions

#### 6.3.1 Emerging pathway-targeted therapies

Several pathway-targeted strategies have been proposed based on findings from COPD, OSA, and experimental models. Nrf2 activation may strengthen endogenous antioxidant and cytoprotective responses, whereas mitochondria-targeted antioxidants may limit reactive oxygen species production and downstream inflammatory signaling (61, 64, 65). Modulation of the NLRP3 inflammasome is another potential approach because mitochondrial stress and cellular injury can promote inflammasome activation and interleukin-1 signaling (66, 67). However, evidence for these strategies is derived largely from COPD and preclinical studies, and their relevance to overlap syndrome remains uncertain. Similar concerns apply to interventions targeting NF-κB or MAPK, whose broad roles in host defense and cellular homeostasis may limit therapeutic specificity. Overlap-specific clinical studies are needed before these approaches can be considered for treatment.

#### 6.3.2 Biomarker-guided and individualized integrated management

COPD-OSA overlap syndrome is clinically and biologically heterogeneous. Some patients may be dominated by severe nocturnal hypoxemia, whereas others may have greater airway inflammation, obesity-related metabolic dysfunction, or cardiovascular comorbidity. Marked airflow limitation, chronic hypercapnia, and frequent exacerbations may further distinguish clinically relevant subgroups. Future management may therefore require more precise phenotyping. Candidate biomarkers of inflammation, oxidative stress, and endothelial injury could be interpreted together with nocturnal oxygen burden, spirometric impairment, exacerbation history, obesity, smoking status, and cardiovascular comorbidities (68). However, no biomarker panel has yet been validated for routine risk stratification or treatment selection. Whether these markers can identify patients who respond differently to positive airway pressure, antioxidant treatment, or anti-inflammatory therapy will need to be tested in prospective, well-phenotyped cohorts (60).

Current management should remain based on established COPD- and OSA-directed therapies, together with treatment of obesity, physical inactivity, smoking, and cardiometabolic comorbidity where appropriate. Antioxidant, anti-inflammatory, mitochondrial, inflammasome-targeted, and biomarker-guided approaches require further overlap-specific clinical evaluation.

## 7 Current challenges and future research priorities

Despite increasing recognition of COPD-OSA overlap syndrome, important uncertainties remain in its mechanisms, phenotypes, and clinical management. Current evidence suggests that chronic airway inflammation, intermittent hypoxia, oxidative stress, and endothelial dysfunction may jointly contribute to pulmonary and systemic injury. However, the literature remains fragmented, and overlap-specific biological and clinical heterogeneity is still insufficiently defined. This limits the translation of mechanistic insights into risk stratification and targeted intervention.

### 7.1 Limitations of current evidence

Interpretation of the current evidence is limited by substantial variation in the definition and assessment of COPD-OSA overlap syndrome. Differences in diagnostic criteria, physiological thresholds, disease severity, cohort characteristics, comparator groups, and biological sample sources reduce comparability across studies and may partly account for inconsistent biomarker findings. Obesity, smoking, infection, cardiometabolic comorbidities, medication use, and adherence to positive airway pressure therapy further complicate interpretation and may also contribute to the variable biomarker responses reported after treatment. In addition, most available studies are small or cross-sectional, and relatively few include parallel COPD-only, OSA-only, and overlap groups. It therefore remains difficult to determine whether the reported abnormalities are specific to overlap syndrome or reflect methodological differences, residual confounding, or the combined burden of the two component disorders.

### 7.2 Unresolved mechanistic questions

Whether COPD-OSA overlap syndrome represents a distinct biological phenotype remains an open question. Greater inflammatory and oxidative activity has been reported in affected patients, but this does not necessarily establish a unique molecular synergy between COPD and OSA. The contribution of endothelial dysfunction, impaired antioxidant defense, mitochondrial injury, HIF-1α signaling, and NLRP3 inflammasome activation may also vary across clinical phenotypes, and much of the supporting evidence is still derived from COPD, OSA, or experimental studies. A further uncertainty is whether biomarker abnormalities contribute directly to disease progression or mainly reflect hypoxemia, airflow limitation, obesity, and systemic comorbidity. Clarifying these relationships will require longitudinal studies with well-defined phenotypes and repeated assessment of biological and clinical outcomes.

### 7.3 Future research priorities

Future studies should use standardized definitions and clearer phenotyping frameworks to improve comparability across cohorts. Longitudinal and multicenter studies with repeated assessment of lung function, sleep-related parameters, nocturnal oxygen burden, biomarker profiles, and comorbidities are needed to clarify disease evolution and identify pathways associated with adverse outcomes. Biomarkers should be interpreted together with physiological and clinical features rather than in isolation. Multi-omics and systems-level approaches may further improve understanding of biological heterogeneity, but their value will depend on whether they can support clinically useful stratification, prognosis, and mechanism-informed intervention. Ultimately, future research should move from descriptive associations toward overlap-specific validation, clinically useful phenotyping, and outcome-focused intervention studies.

## 8 Conclusion

COPD-OSA overlap syndrome is a clinically and biologically heterogeneous condition with substantial pulmonary and systemic consequences. Although the interaction between the two disorders provides a plausible mechanistic framework, most pathway-specific evidence remains indirect, and clinically relevant phenotypes are insufficiently defined. Candidate biomarkers are not yet validated for routine diagnosis, risk stratification, or treatment selection. Future studies should prioritize standardized definitions, longitudinal phenotyping, and outcome-focused validation.
